# Supplementary material for: Single and Joint Associations of Polycyclic Aromatic Hydrocarbon Exposure with Liver Function during Early Pregnancy
Source: Toxics. 2023 Oct 16;11(10):863. doi: 10.3390/toxics11100863 (PMC10610734; doi:10.3390/toxics11100863)
Supplement: Supplementary file 1 [file toxics-11-00863-s001.zip › toxics-2615815-supplementary.pdf]

SUPPLEMENTAL MATERIAL

Single and joint associations of PAHs exposure with liver function during pregnancy: A cross-sectional study

Table S1. The quality control of PAHs assay

Chemical	RT (min)	Precursor Ion	Product Ion	CE (eV)	ISTD	Determination coefficient (R^2)	LOD (μg /L)	Recovery (%)	RSD (%)
1-OHNa-d7	15.987	223	208	15	N/A	N/A	N/A	N/A	N/A
1-OHNap	16.043	216	201	15	1-OHNap-D7	0.9998	0.005	107.05	1.4
2-OHNap	16.547	216	201	15	1-OHNap-D7	0.9999	0.0027	105.71	2.27
9-OHFlu	20.231	254	165	20	1-OHNap-D7	0.9998	0.0023	94.15	2.85
2-OHFlu	22.471	254	239	15	1-OHNap-D7	0.9998	0.0029	91.99	3.18
4-OHPhe	25.209	266	235	25	1-OHNap-D7	0.9999	0.0167	90.08	5.01
9-OHPhe	25.211	266	73	25	1-OHNap-D7	0.9982	0.0044	105.01	4.99
1-OHPhe	25.685	266	73	25	1-OHNap-D7	0.9998	0.0071	81.09	4.96
3-OHPhe	25.854	266	73	25	1-OHNap-D7	0.9999	0.0095	84.5	5.48
2-OHPhe	26.702	266	73	25	1-OHNap-D7	0.9999	0.0115	68.19	2.41
1-OHPyr	30.857	290	73	25	1-OHPyr-D9	0.9999	0.0003	100.3	1.97
1-OHP-d9	31.229	299	73	25	N/A	N/A	N/A	N/A	N/A

Abbreviations: RT, retention time; CE, collision energy; ISTD, internal standard substance; LOD, limit of detection; RSD, relative standard deviation; Internal standard solution: 1-OHNap-d7 and 1-OHP-d9; N/A, not available.

Note: Preparation of internal standard solution: 480ul of 1-OHP-d9 stock solution with a concentration of 0.25mg/ml and 240ul of 1-OHNa-d7 stock solution with a concentration of 5mg/ml were fixed to 24ml with acetonitrile, and the final concentration of 1-OHNa-d7 was 50.9μg/ml, and the final concentration of 1-OHP-d9 was 5μg/ml.

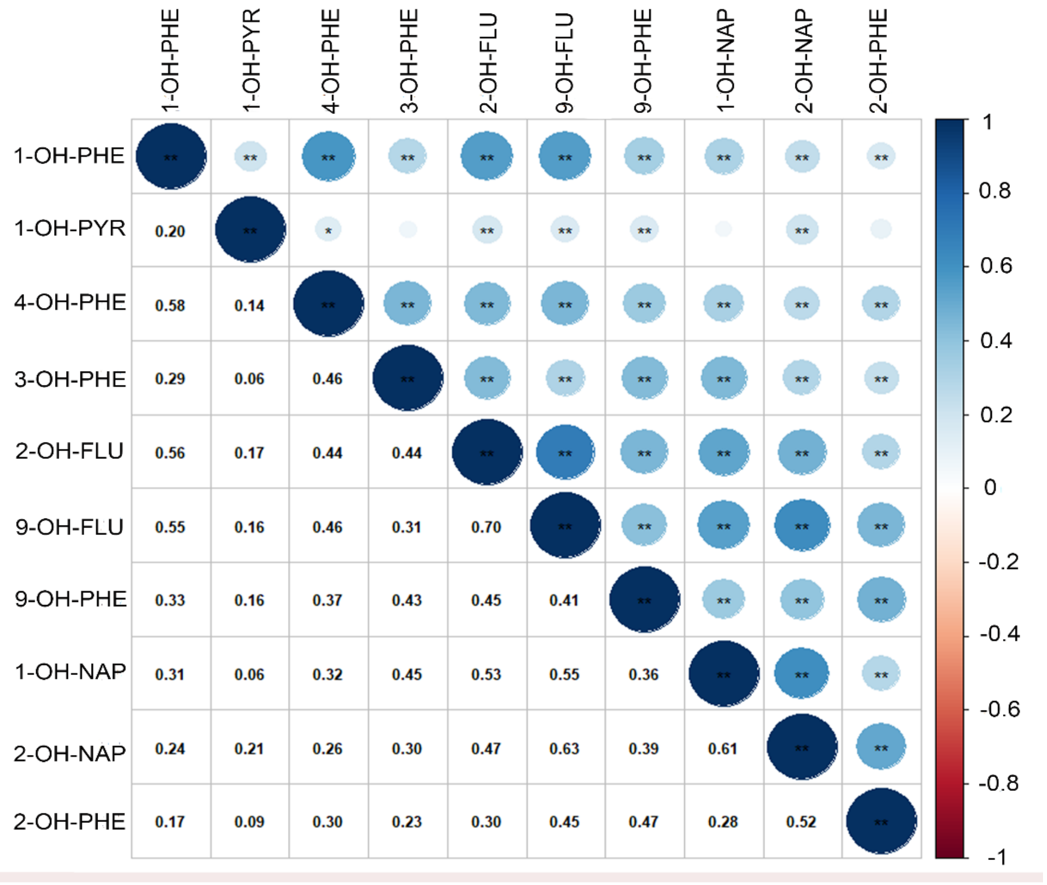

Figure. S1. Pearson correlation analysis of the concentration of PAHs metabolites in the urine of the study population (n=294).
*$P < 0.05$, *$P < 0.01$

Table S2. Relationship between urinary polycyclic aromatic hydrocarbons metabolites and liver function parameters when all chemicals were included in the model.

	ALT		AST	
Analyte (µg/g)	%Δ (95% CI)	P-value	%Δ (95% CI)	P-value
1-OHPHE	-1.29 (-6.48, 4.29)	0.643	-0.70 (-3.34, 2.02)	0.618
2-OHPHE	3.87 (0.30, 7.68)	0.033	2.22 (0.40, 3.98)	0.015
3-OHPHE	0.10 (-4.88, 5.13)	0.997	-0.20 (-2.66, 2.22)	0.863
4-OHPHE	3.46 (-4.11, 11.52)	0.379	1.82 (-1.88, 5.65)	0.338
9-OHPHE	-0.20 (-5.92, 5.97)	0.959	0.60 (-2.27, 3.56)	0.687
2-OHFLU	-3.34 (-11.40, 5.44)	0.441	-2.47 (-6.57, 1.82)	0.261
9-OHFLU	2.33 (-2.96, 7.90)	0.399	1.31 (-1.29, 4.08)	0.314
1-OHNAP	1.01 (-2.08, 4.08)	0.544	0.70 (-0.80, 2.33)	0.346
2-OHNAP	-2.27 (-5.54, 1.01)	0.172	-0.90 (-2.57, 0.70)	0.266
1-OHPYR	-0.30(-2.66, 2.22)	0.834	-0.30 (-1.49, 0.90)	0.627

	ALP		TBA	
	%Δ (95% CI)	P-value	%Δ (95% CI)	P-value
1-OHPHE	0.50 (-3.54, 4.71)	0.822	8.65 (1.11, 16.77)	0.025
2-OHPHE	-1.19 (-3.73, 1.51)	0.391	1.21 (-3.44, 6.08)	0.618
3-OHPHE	1.51 (-2.27, 5.34)	0.439	-3.83 (-9.97, 2.74)	0.243
4-OHPHE	1.01 (-4.59, 6.93)	0.725	3.87 (-6.01, 14.80)	0.457
9-OHPHE	-1.19 (-5.54, 3.25)	0.584	-2.57 (-9.97, 5.34)	0.507
2-OHFLU	1.11 (-5.35, 8.00)	0.748	-9.97 (-19.75, 1.11)	0.075
9-OHFLU	0.10 (-3.83, 4.19)	0.956	4.19 (-2.86, 11.85)	0.247
1-OHNAP	-0.20 (-2.57, 2.12)	0.854	3.87 (-0.30, 8.22)	0.067
2-OHNAP	-1.29 (-3.73, 1.21)	0.317	-4.40 (-8.52, -0.100)	0.047

1-OHPYR	-0.50 (-2.27, 1.41)	0.609	-0.10 (-3.25, 3.15)	0.929

	AST/ALT	
	%Δ (95% CI)	P-value
1-OHPHE	0.70 (-3.05, 4.60)	0.725
2-OHPHE	-1.69 (-4.11, 0.80)	0.183
3-OHPHE	-0.10 (-3.54, 3.46)	0.944
4-OHPHE	-1.69 (-6.76, 3.77)	0.541
9-OHPHE	0.50 (-3.63, 4.81)	0.815
2-OHFLU	1.01 (-4.97, 7.36)	0.743
9-OHFLU	-0.90 (-4.50, 2.94)	0.642
1-OHNAP	-0.40 (-2.57, 1.82)	0.732
2-OHNAP	1.51 (-0.90, 3.98)	0.212
1-OHPYR	0.00 (-1.69, 1.71)	0.966

Abbreviations: ALT, alanine aminotransferase; AST, aspartate aminotransferase; TBA, total bile acid; ALP, alkaline phosphatase

Adjusted for continuous maternal age, pre-pregnancy BMI, categorical education, parity, ethnicity, passive smoking, household income, and Gestational hypertension disorder.

Table S3. Relationship between single polycyclic aromatic hydrocarbon metabolites in urine and liver function parameters (Excluded subjects with urine creatinine concentrations outside the range of 0.3 to 3 g/L, N=287)

	ALT		AST	
Analyte (μg/g)	%Δ (95% CI)	*P*-value	%Δ (95% CI)	*P*-value
1-OHPHE	1.01 (-2.96, 5.13)	0.623	0.90 (-1.09, 2.94)	0.358
2-OHPHE	3.36 (0.40, 86.45)	0.027	2.22 (0.80, 3.77)	0.003
3-OHPHE	0.60 (-3.34, 4.71)	0.755	0.50 (-1.39, 2.53)	0.593
4-OHPHE	3.87 (-1.88, 9.97)	0.191	2.63 (-0.20, 5.55)	0.073
9-OHPHE	1.82 (-2.96, 6.72)	0.466	1.82 (-0.5, 4.19)	0.132
2-OHFLU	0.30 (-5.16, 6.18)	0.909	0.70 (-2.18, 3.56)	0.644
9-OHFLU	1.71 (-1.69, 5.13)	0.334	1.41 (-0.20, 3.15)	0.094
1-OHNAP	0.50 (-1.78, 2.84)	0.683	0.70 (-0.40, 1.82)	0.235
2-OHNAP	-0.20 (-2.57, 2.12)	0.855	0.50 (-0.70, 1.61)	0.427
1-OHPYR	-0.70 (-2.96, 1.61)	0.891	-0.50 (-1.59, 0.70)	0.429

	ALP		TBA	
	%Δ (95% CI)	*P*-value	%Δ (95% CI)	*P*-value
1-OHPHE	-0.10 (-3.05, 2.94)	0.964	7.36 (1.71, 13.20)	0.010
2-OHPHE	-1.69 (-3.82, 0.50)	0.136	0.10 (-3.92, 4.19)	0.977
3-OHPHE	0.40 (-2.47, 3.46)	0.769	-2.57 (-7.69, 2.84)	0.351
4-OHPHE	0.10 (-4.11, 4.39)	0.975	5.97 (-1.88, 14.45)	0.141
9-OHPHE	-1.49 (-4.97, 2.02)	0.395	-1.49 (-7.6, 5.02)	0.650
2-OHFLU	-0.60 (-4.78, 3.67)	0.774	-1.88 (-9.15, 5.97)	0.625
9-OHFLU	-0.80 (-3.25, 1.71)	0.534	2.63 (-1.88, 7.47)	0.257
1-OHNAP	-0.70 (-2.37, 1.01)	0.410	1.21 (-1.88, 4.39)	0.455

2-OHNAP	-1.69 (-3.44, 0.10)	0.051	-1.59 (-4.69, 1.61)	0.318
1-OHPYR	-0.40 (-2.08, 1.31)	0.640	-0.10 (-3.15, 3.05)	0.949

	AST/ALT	
	%Δ (95% CI)	*P*-value
1-OHPHE	-0.10 (-2.86, 2.74)	0.960
2-OHPHE	-1.09 (-3.15, 1.01)	0.303
3-OHPHE	-0.10 (-2.86, 2.74)	0.947
4-OHPHE	-1.19 (-5.07, 2.84)	0.552
9-OHPHE	0.10 (-3.25, 3.36)	0.979
2-OHFLU	0.30 (-3.54, 4.39)	0.868
9-OHFLU	-0.20 (-2.57, 2.12)	0.847
1-OHNAP	0.20 (-1.39, 1.82)	0.795
2-OHNAP	0.70 (-0.9, 2.33)	0.408
1-OHPYR	0.20 (-1.39, 1.82)	0.798

Abbreviations: ALT, alanine aminotransferase; AST, aspartate aminotransferase; TBA, total bile acid; ALP, alkaline phosphatase
Adjusted for continuous maternal age, pre-pregnancy BMI, categorical education, parity, ethnicity, passive smoking, household income, and Gestational hypertension disorder.

Table S4. Posterior inclusion probabilities (PIPs) for each urinary PAHs metabolites included in the BKMR models.

Analyte	Group	ALT		AST		TBA	
		groupPIP	condPIP	groupPIP	condPIP	groupPIP	condPIP
1-OH-PHE	1	0.3010	0.0558	0.3248	0.0025	0.7778	0.8218
2-OH-PHE	1	0.3010	0.4525	0.3248	0.8947	0.7778	0.0111
3-OH-PHE	1	0.3010	0.0538	0.3248	0.0012	0.7778	0.0206
4-OH-PHE	1	0.3010	0.1003	0.3248	0.0333	0.7778	0.0540
9-OH-PHE	1	0.3010	0.0764	0.3248	0.0191	0.7778	0.0103
2-OH-FLU	2	0.1296	0.2963	0.0274	0.1022	0.5666	0.7656
9-OH-FLU	2	0.1296	0.7037	0.0274	0.8978	0.5666	0.2344
1-OH-NAP	1	0.3010	0.1037	0.3248	0.0289	0.7778	0.0224
2-OH-NAP	1	0.3010	0.0711	0.3248	0.0080	0.7778	0.0414
1-OH-PYR	1	0.3010	0.0864	0.3248	0.0123	0.7778	0.0185

Abbreviations: ALT, alanine aminotransferase; AST, aspartate aminotransferase; TBA, total bile acid

BKMR models were adjusted for continuous maternal age, pre-pregnancy BMI, categorical education, ethnicity, nation, passive smoking, household income, and Gestational hypertension disorder.

condPIP: The posterior probabilities of particular PAH exposure within their according to groups from the multiple iterations (50,000) of the MICMIC sampler

groupPIP: The posterior probabilities of exposure-included groups from the multiple iterations (50.000) of the MCMC sampler.

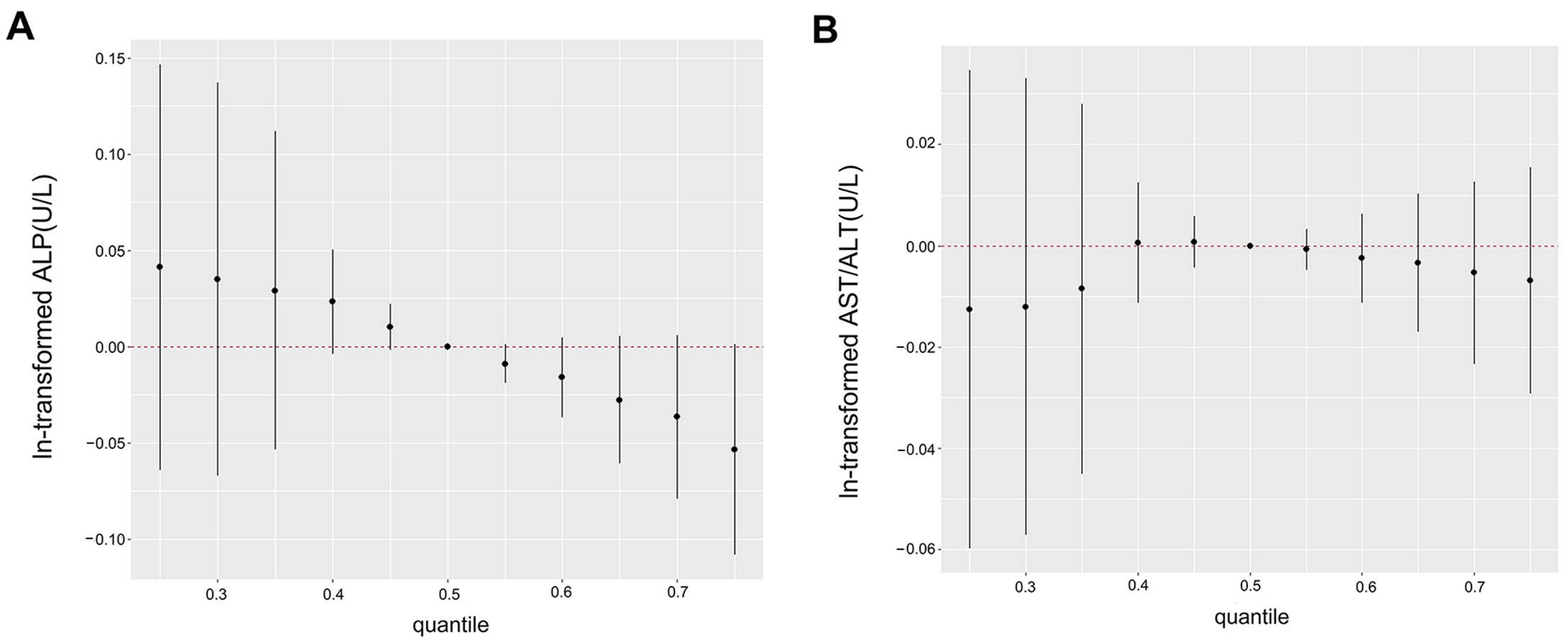

Figure. S2. The joint associations of the PAHs mixture on the ALP (A), AST/ALT (B) levels were estimated by Bayesian Kernel Machine Regression (BKMR). Adjusted for continuous maternal age, pre-pregnancy BMI, categorical education, parity, ethnicity, passive smoking, household income, and hypertension.

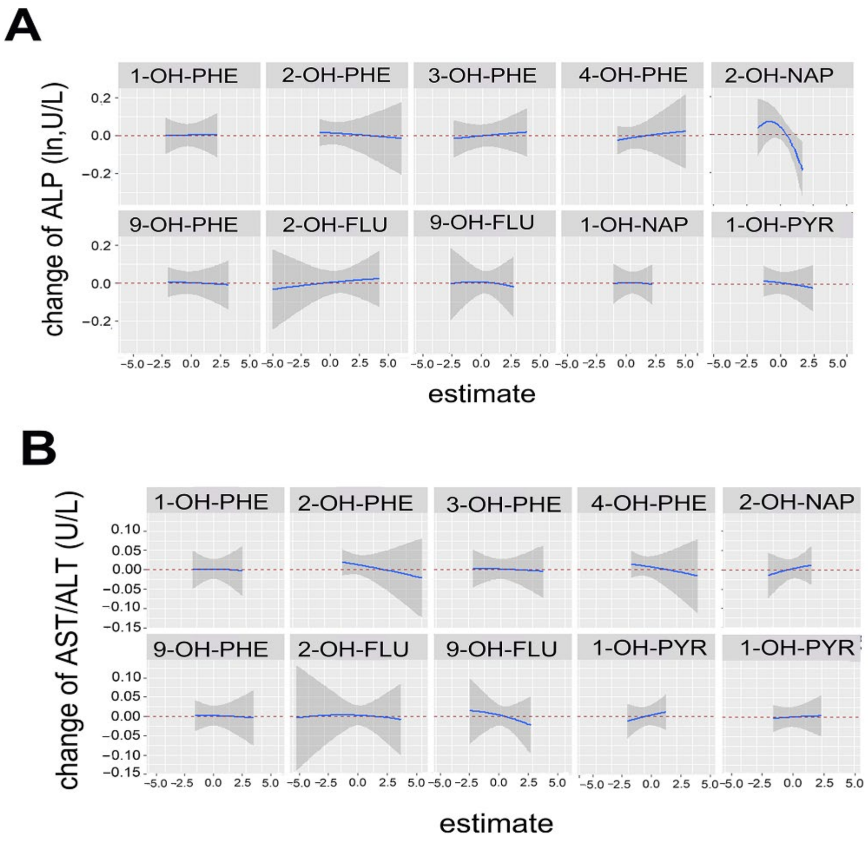

Figure. S3. Univariate exposure-response relationship between the concentration of each substance ALP (A), AST/ALT (B) parameter when other substances are fixed at the median concentration. The model adjusted for continuous maternal age, pre-pregnancy BMI, ethnicity, categorical education, parity, passive smoking, household income, and hypertension.

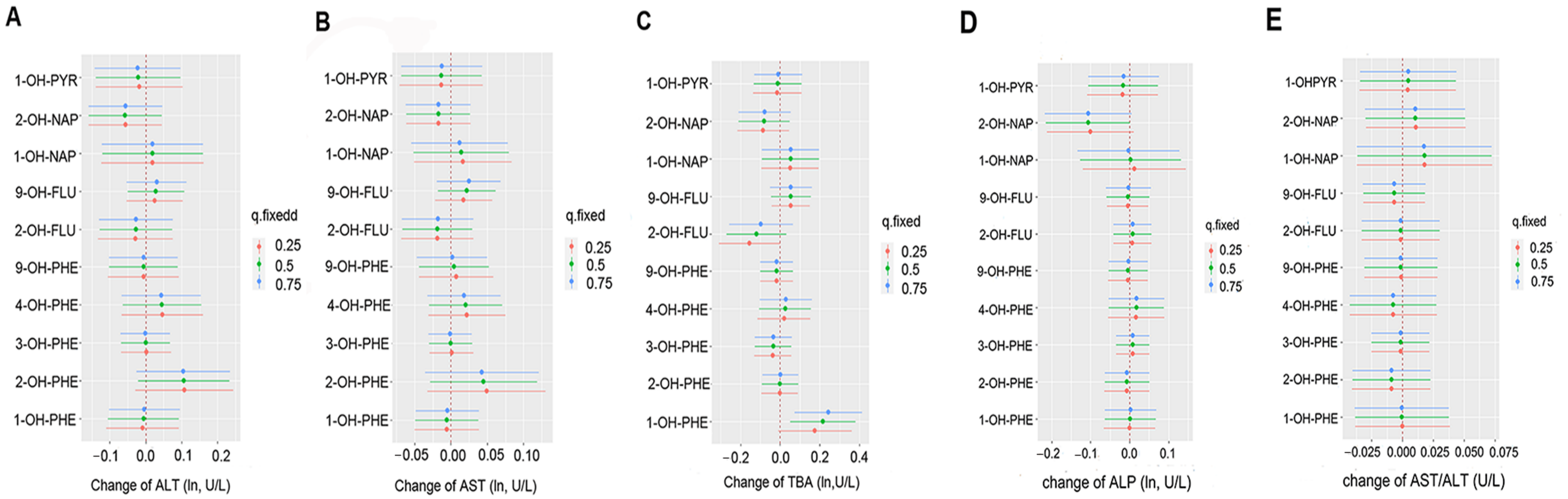

Figure. S4. The association between increased IQR per substance concentration and liver function parameters when other substances were fixed at the 25th, 50th, or 75th percentile, respectively. Adjusted for continuous maternal age, pre-pregnancy BMI, categorical education, ethnicity, nation, passive smoking, household income, and Gestational hypertension disorder.
